# Peripheral nervous system involvement accompanies central nervous system involvement in anti-glial fibrillary acidic protein (GFAP) antibody-related disease

**DOI:** 10.1007/s00415-023-11908-7

**Published:** 2023-08-04

**Authors:** Julian Theuriet, Florent Cluse, Alice Gravier-Dumonceau, Géraldine Picard, Sterenn Closs, Véronique Rogemond, Noémie Timestit, Françoise Bouhour, Philippe Petiot, Vincent Davy, Eve Chanson, Joaquín Arzalluz-Luque, Romain Marignier, Jerome Honnorat, Antoine Pegat

**Affiliations:** 1 Service d’electroneuromyographie et de pathologies neuromusculaires, Hôpital Neurologique Pierre Wertheimer, Hospices Civils de Lyon, Groupement Est, 59 boulevard Pinel, Bron, France; 2 Service de Neurologie C, troubles du mouvement et pathologies neuromusculaires, Hôpital Neurologique Pierre Wertheimer, Hospices Civils de Lyon, Groupement Est, Bron, France; 3 French Reference Centre on Paraneoplastic Neurological Syndrome, Hôpital Neurologique Pierre Wertheimer, Hospices Civils de Lyon, Bron, France; 4 Service de biostatistique, Hospices Civils de Lyon, Lyon, France; 5 Service de neurologie, Hôpital Pitié Salpétrière, Assistance Publique des Hôpitaux de Paris, Paris, France; 6 Service de neurologie, CHU de Clermont-Ferrand, Clermont-Ferrand, France; 7 Service de Neurologie, sclérose en plaques, pathologies de la myéline et neuro-inflammation, Hôpital Neurologique Pierre-Wertheimer, Hospices Civils de Lyon, Bron, France; 8 Centre de Référence des Maladies Inflammatoires Rares du Cerveau et de la Moelle (MIRCEM), Hôpital Neurologique Pierre-Wertheimer, Hospices Civils de Lyon, Bron, France; 9 MeLiS-UCBL-CNRS UMR 5284-INSERM U1314, Université Claude Bernard Lyon 1, Lyon, France

**Keywords:** GFAP, Peripheral neuropathy, Autoimmune encephalitis, ENMG

## Abstract

**Background:**

Glial fibrillary acidic protein (GFAP) is expressed by astrocytes in the central nervous system (CNS), but also by immature and regenerative Schwann cells in the peripheral nervous system (PNS). GFAP antibodies (GFAP-Abs) in cerebrospinal fluid (CSF) have been mainly described in patients with meningoencephalomyelitis. We aimed to study PNS symptoms in patients with CSF GFAP-Abs.

**Methods:**

We retrospectively included all patients tested positive for GFAP-Abs in the CSF by immunohistochemistry and confirmed by cell-based assay expressing human GFAPα since 2017, from two French reference centers.

**Results:**

In a cohort of 103 CSF GFAP-Abs patients, 25 (24%) presented with PNS involvement. Among them, the median age at onset was 48 years and 14/25 (56%) were female. Abnormal electroneuromyography was observed in 11/25 patients (44%), including eight isolated radiculopathies, one radiculopathy associated with polyneuropathy, one radiculopathy associated with sensory neuronopathy, and one demyelinating polyradiculoneuropathy. Cranial nerve involvement was observed in 18/25 patients (72%). All patients except one had an associated CNS involvement. The first manifestation of the disease concerned the PNS in three patients. First-line immunotherapy was administered to 18/24 patients (75%). The last follow-up modified Rankin Scale was ≤ 2 in 19/23 patients (83%). Patients with PNS involvement had significantly more bladder dysfunction than patients with isolated CNS involvement (68 vs 40.3%, *p* = 0.031).

**Conclusions:**

PNS involvement in GFAP-Abs autoimmunity is heterogeneous but not rare and is mostly represented by acute or subacute cranial nerve injury and/or lower limb radiculopathy. Rarely, PNS involvement can be the first manifestation revealing the disease.

**Supplementary Information:**

The online version contains supplementary material available at 10.1007/s00415-023-11908-7.

## Introduction

Glial fibrillary acidic protein (GFAP) is the predominant neurofilament in the astrocytes of the central nervous system (CNS), responsible for the cytoskeleton structure [1]. GFAP has been identified as an antigenic target of an inflammatory CNS disease, called autoimmune anti-GFAP astrocytopathy [2]. GFAP is also expressed in the peripheral nervous system (PNS) in non-myelinating immature Schwann cells and in satellite cells of dorsal root ganglia (DRG). It is likely, however, that CNS and PNS GFAP are not perfectly identical, since a monoclonal GFAP antibody was found to selectively recognize GFAP in astrocytes and not in PNS cells [3–5]. Furthermore, some studies have shown the expression of this protein in Schwann cells after axonal damage, and a poor Schwann cell proliferation is observed after nerve injury when the GFAP expression is lost [6, 7]. *GFAP* mutations are also associated with demyelinating neuropathies in Alexander disease [8]. All these data suggest that GFAP could be a target in some PNS autoimmune diseases. Six patients with PNS involvement and autoimmune anti-GFAP astrocytopathy have been reported, and PNS symptoms were the initial manifestation for five of them [9]. Interestingly, a T-cell predominant perivascular inflammatory infiltrate and demyelinating features were observed in the nerve biopsies of these patients [9]. However, the clinical presentation seems to be heterogeneous, since polyradiculoneuropathy [9], radiculoneuritis [10], axonal sensorimotor neuropathy [11], axonal predominant motor neuropathy [12], and acute motor axonal neuropathy (AMAN) with positive GM1 IgG antibodies [13] have been reported. Despite these few reports, the phenotype of PNS involvement in autoimmune anti-GFAP astrocytopathy remains unclear, while the central manifestations have been subsequently characterized by several cohorts [14–17]. The aim of this study was to describe PNS involvement in a cohort of patients with cerebrospinal fluid (CSF) GFAP antibodies (GFAP-Abs).

## Methods

### Study participants and assays

All patients tested positive for CSF GFAPα-IgG-Abs between May 2017 and June 2022 in the Reference Center for Rare Brain and Spinal Cord Inflammatory Diseases (MIRCEM) and in the French National Reference Center for Paraneoplastic Neurological Syndromes both in Lyon, France, were included. Regarding the assays, an indirect immunofluorescence assay (IFA) was used for the screening of GFAP-Abs in the patients’ CSF. As previously described, those with a compatible fluorescence positivity were tested by cell-based assay (CBA) for confirmation [14]. When available, the serum samples, which were taken concomitantly to CSF samples, were tested using CBA.

Data of interest were retrospectively collected from the treating physicians using a structured questionnaire. It included demographic data, clinical features, electroneuromyography (ENMG) with nerve conduction studies and needle electromyography, CSF study, brain and spinal cord MRI features, admission to intensive care unit (ICU), modified Rankin scale (mRS) at diagnosis and last follow-up, first-line and long-course immunotherapy, the final preferred diagnosis retained by the clinicians in charge of the patients, and the alternative differential diagnoses that were still being considered at last examination by these clinicians. PNS involvement was defined by both clinical and electrophysiological evidence for peripheral neuropathies or radiculopathies, and by clinical evidence for cranial nerve injury. The EAN/PNS 2021 criteria of chronic inflammatory demyelinating polyradiculoneuropathies (CIDP) were used to define demyelinating polyradiculoneuropathies, and the criteria of Camdessanche et al*.* were used to define sensory neuronopathies [18, 19]. Results from ENMG were reviewed by three neurologists (J.T., F.C., and A.P.).

Acute and subacute onset were defined as a time frame of 3 months or less between symptom onset and peak, and progressive onset was defined as a time frame of more than 3 months between symptom onset and peak. To assess treatment outcome, significant response was defined as a decrease of at least two points on the mRS or full recovery.

In a final step, the more relevant clinical data of patients with PNS involvement (sex, age of onset, mode of onset, frequency of lower limb weakness, type of CNS involvement, areflexia, bladder dysfunction, maximal mRS, and last-follow-up mRS) were compared with those of patients with an isolated CNS involvement.

### Statistical analyses

The description of the data was made through the use of appropriate quantitative data: median (quartiles Q1/Q3) for the continuous variables, and proportions for the categorical variables. The comparison of each variable of interest between the group with PNS involvement vs the group with isolated CNS involvement was made using appropriate statistical tests: Chi2 (or Fisher’s exact if the conditions were not met) for the categorical variables and Student (or Wilcoxon if the conditions were not met) for continuous variables. An alpha risk of 5% was considered bilaterally. Missing data were not computed. All statistical analyses were done using R version 4.0.3 (R Foundation for Statistical Computing, Vienna, Austria).

### Standard protocol approvals, registrations, and patient consents

All patients received oral and written information regarding the study. All procedures in this study were carried out in accordance with the ethical standards of the *Hospices Civils de Lyon* (ethics approval 22_5018) and with the 1964 Helsinki declaration. Human biological samples were stored in NeuroBioTec (CRB HCL, Lyon France, Biobank BB-0033-00046).

## Results

### Demographic features of patients with CSF GFAP-Abs and PNS involvement

**Fig. 1:**
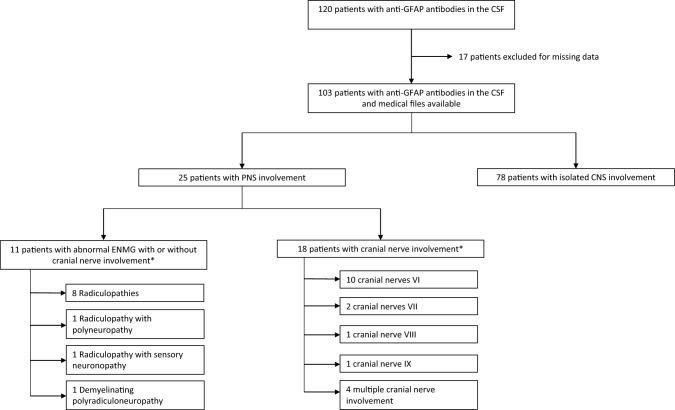
Diagram of patients with CSF GFAP antibodies and peripheral nervous system involvement. CSF, cerebrospinal fluid; CNS, central nervous system; PNS, peripheral nervous system; ENMG, electroneuromyography. *4 patients presented with both pathological ENMG and cranial nerve involvement

**Fig. 2:**
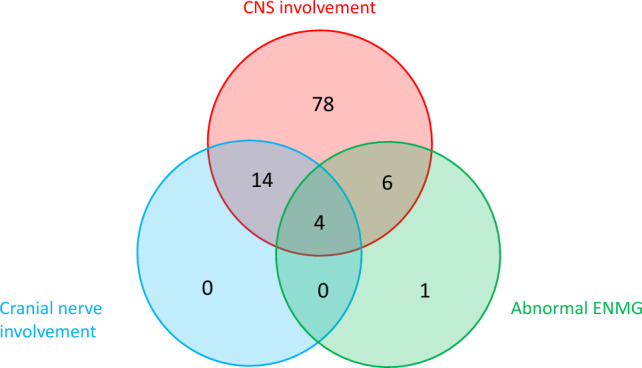
Venn diagram representing patients’ repartition according to CNS involvement, cranial nerve involvement, and abnormal ENMG. CNS, central nervous system; ENMG, electroneuromyography

From May 2017 to June 2022, 120 patients were tested positive for CSF GFAP-Abs in the two French reference centers (Fig. 1). Due to a lack of clinical and paraclinical data preventing us to determine the presence or absence of PNS involvement, 17 patients were excluded; 103 were finally included. Isolated involvement of the CNS was observed in 78 patients (76%), while 25 (24%) presented with PNS involvement and were finally analyzed herein (Fig. 1). All 25 patients except one (patient 9, with isolated meningoradiculitis) had associated CNS involvement (Fig. 2). In the group of 25 patients with PNS involvement, the median age at disease onset was 48 years (IQR: 29–60), 14 patients were female (56%), 23 (96%) presented with acute or subacute disease onset, and one had progressive disease (the mode of onset was unknown for one). Neoplasms were found in three patients (12%), including two ovarian teratomas and one lung adenocarcinoma. All tumors were detected subsequently to neurological symptoms, due to specific screenings (Table 1). Patient 6, with radiculopathy associated with sensory neuronopathy, had a lung adenocarcinoma identified during neoplasm screening, but onco-neuronal antibodies were negative (Table 2). Among the 25 patients with PNS involvement, patient 11 was the only one with an associated autoimmune disease (psoriasis), and patient 6 was the only one with a past medical history that could cause PNS damage (type 2 diabetes mellitus), but without PNS symptoms before CNS involvement.

**Table 1:** Clinical, paraclinical, and treatment characteristics of patients with PNS involvement and CSF GFAP antibodies

	Number of patients (%)	Median (IQR)
Age at onset, years		48 (29–60)
Female sex	14/25 (56)	
Cranial nerve involvement^a^	18/25 (72)	
Abnormal ENMG^b^	11/25 (44)	
Radiculopathy	10/11	
Demyelinating polyradiculoneuropathy	1/11	
Sensory neuronopathy	1/11	
Polyneuropathy	1/11	
Onset		
Acute and subacute	23/24 (96)	
Progressive	1/24 (4)	
CNS involvement at onset	22/25 (88)	
PNS involvement at onset	3/25 (12)	
CNS involvement	24/25 (96)	
Meningoencephilitis^c^	13/24 (54)	
Meningoencephalomyelitis	6/24 (25)	
Meningitis^d^	5/24 (21)	
Tumor revealed at screening	3/25 (12)	
CSF findings		
Pleocytosis	25/25 (100)	
White cell count (/mm^3^)		143 (93–285.75)
Elevated proteins	25/25 (100)	
Protein level (g/L)		1.07 (0.8–1.42)
Hypoglycorrhachia	2/20 (10)	
Oligoclonal bands (≥ 2)	12/15 (80)	
Brain MRI		
Leptomeningeal enhancement	12/23 (52)	
Cranial nerve enhancement	7/23 (30)	
Spinal cord MRI		
Leptomeningeal enhancement	13/18 (72)	
Root enhancement	5/18 (28)	
Admission to ICU	9/25 (36)	
Maximal mRS score		4 (2–5)
0–2	7/25 (28)	
3–5	18/25 (72)	
6	0/25 (0)	
Duration of follow-up, months		12 (4–12)
First-line immunotherapy	18/24 (75)	
Corticosteroids	15/24 (63)	
IVIg	9/24 (38)	
PLEX	6/24 (25)	
Rituximab	1/24 (4)	
Response to first-line immunotherapy	16/18 (89)	
Long-course treatment	16/23 (70)	
Oral corticosteroids	12/23 (52)	
IVIg	4/23 (17)	
PLEX	2/23 (9)	
Rituximab	3/23 (13)	
mRS at last follow-up		1 (1–2)
0–2	19/23 (83)	
3–5	4/23 (17)	
6	0/19 (0)	

GFAP, glial fibrillary acidic protein; ENMG, electroneuromyography; CNS, central nervous system; CSF, cerebrospinal fluid; mRS, modified Rankin Scale; IVIg, intravenous immunoglobulin; PLEX, plasma exchange; ICU, intensive care unit

^a^Four patients had cranial nerve involvement and abnormal ENMG

^b^Two patients had two abnormalities on ENMG (one with sensory neuronopathy and radiculopathy and one with polyneuropathy and radiculopathy)

^c^Including two patients with rhomboencephalitis

^d^With clinical signs of meningeal syndrome

**Table 2:** Clinical and paraclinical characteristics of patients with CSF GFAP antibodies and abnormal ENMG

Patient	Sex (M/F)/age at onset (years)	First system involved (CNS/PNS)	Type of CNS involvement	Type of PNS involvement	Cranial nerve involvement	Limb weakness	Sensory signs	CSF			Leptomeningeal or root/cranial nerve enhancement on MRI	Preferred final diagnosis	Alternative differential diagnosis
								White cell count	Protein level	Oligoclonal bands (≥ 2)			
1	M/62	CNS	Meningoencephalitis	LL radiculopathy + polyneuropathy	–	–	+ (LL paresthesia and apallaesthesia)	35	0.71	+	+ (spinal leptomeninges)	GFAP-Abs-related disease	/
2	F/33	CNS	Meningoencephalitis	LL radiculopathy	–	+ (LL, P)	+ (LL paresthesia)	171	1.49	+	+ (spinal and brain leptomeninges, roots)	GFAP-Abs-related disease	/
3	M/84	CNS	Meningitis	Demyelinating polyradiculoneuropathy	+ (VI)	+ (LL, P)	–	33	0.58	+	–	GFAP-Abs-related disease	/
4	M/54	CNS	Meningoencephalomyelitis	LL radiculopathy	–	+ (LL, P)	+ (LL paresthesia, hypoesthesia and apallesthesia)	250	2.2	NA	+ (spinal leptomeninges, cranial nerves)	GFAP-Abs-related disease	/
5	F/47	CNS	Meningoencephalomyelitis	LL radiculopathy	–	+ (LL, P)	+ (LL paresthesia and hypo-pallaesthesia, radiculalgia)	100	0.97	+	–	GFAP-Abs-related disease	/
6	M/69	CNS	Meningitis	LL radiculopathy + Sensory neuronopathy	+ (VI)	+ (LL, P)	+ (LL distal apallesthesia, radiculalgia)	50	0.8	+	–	GFAP-Abs-related disease	/
7	F/22	CNS	Meningoencephalomyelitis	LL radiculopathy	+ (IX, X)	+ (LL, P)	–	750	1.34	+	+ (brain and spinal leptomeninges, roots)	GFAP-Abs-related disease	/
8	M/29	CNS	Meningoencephalitis	LL radiculopathy	–	+ (LL, P and D)	–	NA	3.5	NA	+ (brain and spinal leptomeninges, cranial nerves)	GFAP-Abs-related disease	Tuberculosis
9	F/56	PNS	/	LL radiculopathy	–	+ (LL, D)	+ (LL radiculalgia)	71	0.99	+	+ (spinal leptomeninges and roots)	Lyme meningoradiculitis	GFAP-Abs-related disease
10	M/46	CNS	Meningoencephalomyelitis	LL radiculopathy	+ (VIII)	–	–	100	1.56	NA	+ (brain and spinal leptomeninges, cranial nerves)	GFAP-Abs-related disease triggered by VZV	/
11	M/58	CNS/PNS	Meningoencephalitis	LL radiculopathy	–	–	–	72	2.18	NA	+ (spinal leptomeninges, roots)	GFAP-Abs-related disease triggered by Lyme disease	/
12	F/23	CNS	Meningoencephalitis	/	+ (VI)	–	–	509	1.42	–	+ (brain leptomeninges)	GFAP-Abs-related disease	/
13	F/73	CNS	Meningitis	/	+ (VII)	–	–	193	0.58	NA	–	GFAP-Abs-related disease	/
14	M/53	CNS	Meningoencephalitis	/	+ (VI)	–	–	112	1.25	–	–	GFAP-Abs-related disease	/
15	F/35	CNS	Meningoencephalitis	/	+ (VI)	–	–	1390	1.39	+	+ (brain leptomeninges)	GFAP-Abs-related disease	/
16	F/26	CNS	Meningoencephalitis	/	+ (VII)	+ (UL, LL)	–	281	0.5	–	–	GFAP-Abs-related disease	/
17	F/19	CNS	Meningoencephalitis	/	+ (VI)	–	–	150	0.89	NA	+ (spinal leptomeninges)	GFAP-Abs-related disease	/
18	M/60	CNS	Meningoencephalitis	/	+ (IX)	+ (UL, LL, PD)	–	110	0.94	NA	+ (brain leptomeninges)	GFAP-Abs-related disease	/
19	M/37	PNS	Meningoencephalitis	/	+ (V, VII, IX, X, XI)	–	+ (neuropathic pain)	300	0.55	+	+ (brain leptomeninges)	GFAP-Abs-related disease	/
20	F/63	CNS	Meningoencephalomyelitis	/	+ (V, VII)	+ (LL, PD)	+ ( LL hypoesthesia and apallesthesia)	71	1.34	+	+ (brain and spinal leptomeninges, cranial nerves)	GFAP-Abs-related disease	/
21	M/55	CNS	Meningitis	/	+ (VI)	–	–	229	1.1	NA	+ (brain leptomeninges, cranial nerves)	GFAP-Abs-related disease	/
22	F/48	CNS	Meningoencephalomyelitis	/	+ (VI)	+ (UL, LL, PD)	–	100	0.6	+	+ (brain and spinal leptomeninges, cranial nerves and roots)	GFAP-Abs-related disease	/
23	F/21	CNS	Meningoencephalitis	/	+ (VI)	–	–	420	1.07	NA	–	GFAP-Abs-related disease	/
24	F/16	CNS	Meningoencephalomyelitis	/	+ (VI)	+ (LL, PD)	–	115	0.9	NA	+ (brain and spinal leptomeninges, cranial nerves)	GFAP-Abs-related disease	/
25	F/63	CNS	Meningoencephalitis	/	+ (VI, VII)	–	–	205	1.46	+	+ (spinal leptomeninges)	GFAP-Abs-related disease	/

GFAP, glial fibrillary acidic protein; Abs, antibodies; CNS, central nervous system; CSF, cerebrospinal fluid; PNS, peripheral nervous system; M, male; F, female; LL, lower limbs; UL, upper limbs; P, proximal; D, distal; VZV, varicella-zoster virus; NMOSD, neuromyelitis optica spectrum disorders; NA, not available

### Clinical and electrophysiological characteristics of patients with CSF GFAP-Abs and PNS involvement

The first neurological manifestation was peripheral for three patients (12%), two with lower limb meningoradiculitis (patients 9 and 11), and one with trigeminal neuralgia (patient 19). The first one (patient 9) did not develop CNS involvement during the course of the disease. The second (patient 11) rapidly developed hiccups suggestive of area postrema syndrome and bilateral papilledema without elevated opening pressure on lumbar puncture, while the last one (patient 19) rapidly developed signs of rhombencephalitis (facial diplegia, dysphagia, and bulbar FLAIR hyperintensity on brain MRI). The other 22 patients (88%) started their disease with CNS involvement as the first neurological manifestation (Table 1).

Eleven patients (44%) had pathological ENMG. Lower limb weakness was observed in eight of them, with a proximal predominance for six, including five with radiculitis. Six of these patients presented with sensory symptoms/signs in the lower limbs, such as paresthesia, hypoesthesia, and apallesthesia. Radicular pain was observed in three patients. Details concerning their clinical characteristics are given in Table 2. Regarding the ENMG results: eight patients had isolated radiculopathy, one had radiculopathy associated with sensory neuronopathy, one had radiculopathy associated with length-dependent axonal polyneuropathy, and one had demyelinating polyradiculoneuropathy (Tables 1 and 3). Prolonged F-wave latencies were the most common feature, present in nine patients. A neurogenic pattern on needle examination was observed in only three patients, associated with fibrillation or positive sharp waves. Follow-up ENMG improved in 5/8 patients with available follow-up data. Details concerning the ENMG are given in Table 3.

**Table 3:** Electrophysiological findings of patients with CSF GFAP antibodies and abnormal ENMG

Patient	Type of PNS involvement	CMAP amplitude	F-wave latencies	Fibrillation potentials and/or positive sharp waves	Contraction pattern	Improvement in ENMG (follow-up duration, months)
1	Radiculopathy + Polyneuropathy	Decreased	Prolonged	No	Normal	NA
2	Radiculopathy	Normal	Prolonged	Yes	Neurogenic	Yes (1)
3	Demyelinating polyradiculoneuropathy	Decreased	Prolonged	No	Normal	Yes (36)
4	Radiculopathy	Decreased	Prolonged	Yes	Neurogenic	Yes (9)
5	Radiculopathy	Normal	Prolonged	No	Normal	NA
6	Radiculopathy + sensory neuronopathy	Decreased	Normal	No	Normal	No (24)
7	Radiculopathy	Normal	Prolonged	No	Normal	NA
8	Radiculopathy	Normal	Prolonged	No	Normal	Yes (12)
9	Radiculopathy	Decreased	Normal	Yes	Neurogenic	No (2)
10	Radiculopathy	Decreased	Prolonged	No	Normal	No (0.5)
11	Radiculopathy	Normal	Prolonged	No	Normal	Yes (3)

CMAP, compound muscle action potential; ENMG, electroneuromyography; NA, not available; CSF, cerebrospinal fluid; PNS, peripheral nervous system; VZV, varicella-zoster virus

Eighteen patients (72%) demonstrated cranial nerve involvement (Fig. 1, Table 2). Cranial nerve injury was without other signs of neuropathy in 14 patients (55%), while four had both limb and cranial nerve involvement. ENMG was abnormal in 4 of these 14 patients, and not performed in the remaining ten patients in the absence of clinical suspicion of limb neuropathy. Nerve VI was the most frequently affected, occurring in ten patients (45%). Papilledema was observed in four of them (40%). Nerve VII was affected in two patients (8%). Nerves VIII and IX were also found to be affected in one patient for each (4%), while four patients (16%) experienced multiple cranial nerve involvement (including the one who presented with the trigeminal neuralgia as first symptom).

### Serological, CSF, MRI characteristics, and final diagnoses of patients with CSF GFAP-Abs and PNS involvement

**Fig. 3:**
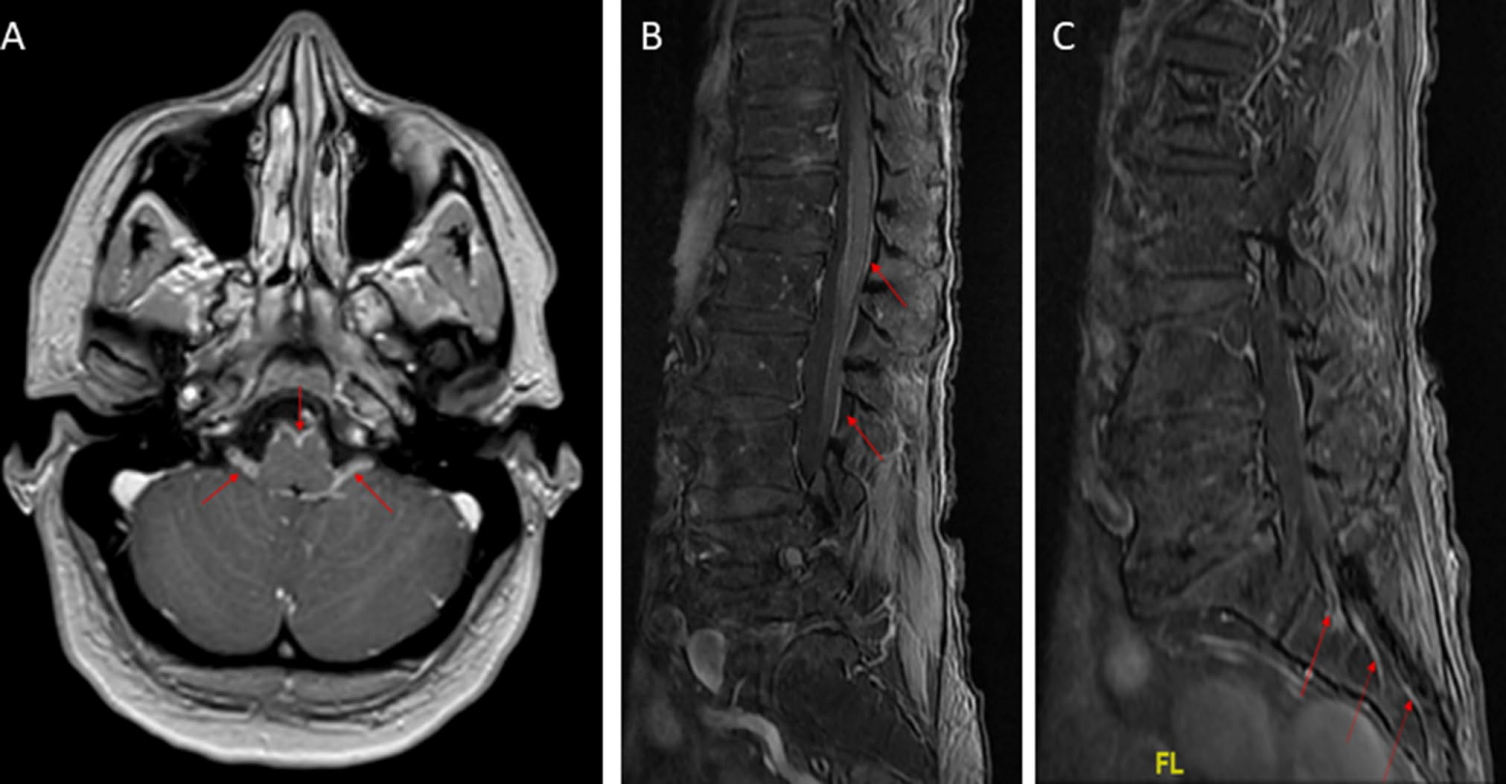
Brain and spinal cord MRI features of patients with CSF GFAP antibodies and PNS involvement. Brain MRI of a patient with glial fibrillary acidic protein (GFAP) antibodies showing brainstem leptomeningeal and acoustic-facial bundle contrast enhancement (**A**, red arrows). Spinal cord MRI of a patient with GFAP antibodies showing medullar leptomeningeal and root contrast enhancement (**B**, **C**, red arrows)

All patients had elevated white cell count in the CSF with a median of 143/mm^3^ (IQR: 93–285.75), and elevated protein level with a median of 1.07 g/L (IQR: 0.8–1.42). Hypoglycorrhachia was observed in 2/20 patients (10%). Oligoclonal bands were found in the CSF of 12/15 tested patients (80%). Brain and spinal cord MRI results were available in 23 and 18 patients, respectively. Twelve patients (52%) had abnormal leptomeningeal gadolinium enhancement on brain MRI, and cranial nerve enhancement was found in seven patients (30%). Concerning spinal cord MRI, an abnormal leptomeningeal enhancement was observed in 13 patients (72%) and root enhancement in 5 patients (28%; Table 1, Fig. 3). Only 2/11 (18%) patients with pathological ENMG had a spinal cord MRI without gadolinium enhancement (Table 2). Anti-gangliosides (*n* = 2), anti-Hu (*n* = 25), and anti-CV2/CRMP5 antibodies (*n* = 25) were not detected in all tested patients. Only 1/18 patient had co-existing antinuclear antibodies, without clinical signs of connective tissue disease.

The preferred final diagnosis retained by the clinicians in charge of the patients was a GFAP-Abs-related disease in 24/25 patients (96%). Two of these patients with radiculopathy had serological signs of recent infection. One had Lyme antibodies (patient 11) in the CSF and another had a CSF positive PCR for the varicella-zona virus (VZV, patient 10; Tables 2 and 3). They were finally considered by the clinicians in charge as GFAP-Abs-related disease triggered by infections. Only patient 9, who also had Lyme antibodies in the CSF, was considered as having a Lyme disease with GFAP-Abs considered as an alternative diagnosis (Tables 2 and 3). The remaining 22 patients had no identified infectious agents in the CSF despite viral, bacterial, and Lyme antibody screenings. Patient 8, who had a radiculopathy and was initially seen by infectious disease specialists, benefited from a full-length anti-tuberculosis treatment, even though the PCR and specific culture were negative, and despite the fact that GFAP-Abs-related disease was retained as the final diagnosis.

Seven patients were concomitantly tested negative for GFAP-Abs in the serum (four patients with isolated cranial nerve involvement, one with inflammatory demyelinating polyradiculoneuropathy, one patient with sensory neuronopathy and radiculopathy, and one patient with radiculopathy and cranial nerve involvement).

### Treatment and outcome of patients with CSF GFAP-Abs and PNS involvement

Nine patients (36%) required admission to ICU. Eighteen patients (72%) evolved toward a maximum mRS of 3 or more, indicating severe impairment, while seven (28%) had a maximum mRS of 0–2. The median follow-up of the 25 patients with PNS involvement was 12 months (IQR: 4–12). Eighteen patients (75%) were treated with first-line immunotherapy as an attack treatment, including corticosteroids (IV for all except one, *n* = 15), intravenous immunoglobulin (IVIg, *n* = 9), plasma exchange (PLEX, *n* = 6), and rituximab (*n* = 1). These treatments were combined in eight patients (56%). A significant response was observed in 16/18 patients (89%). Long-course therapy was maintained in 16 of 23 patients (70%), including oral corticosteroids (*n* = 12), IVIg (*n* = 4), PLEX (*n* = 2), and rituximab (*n* = 3). Nineteen of 23 patients (83%) presented a good clinical outcome characterized by a last follow-up mRS of 0–2. Only four patients (17%) had a last follow-up mRS of 3 or more, and no patient died (Table 1).

### Comparison between patients with PNS involvement and patients with isolated CNS involvement

Bladder dysfunction was significantly more frequent in the patients with PNS involvement (68 vs 40.3%, *p* = 0.031). Areflexia did not differ significantly between the two groups but tended to be more frequent in the group with PNS involvement (32 vs 11.9%, *p* = 0.059). Sex, age of onset, mode of onset, frequency of lower limb weakness, presence of an associated tumor, and type of CNS involvement did not differ significantly between the groups. Regarding clinical severity and prognosis, there was no significant difference in maximal mRS or last-follow-up mRS between the groups. Importantly, the median follow-up did not differ significantly between the two groups (Table 4).

**Table 4:** Comparison between patients with CSF GFAP-Abs and PNS involvement and those with isolated CNS involvement

	PNS ± CNS	CNS	*p* value	Total
	*n* = 25	*n* = 78		*n* = 103
Sex				
Female	14 (56.0%)	32 (41.0%)	0.280	46 (44.7%)
Male	11 (44.0%)	46 (59.0%)		57 (55.3%)
	*n* = 25	*n* = 78		*n* = 103
Age at onset, years				
Median	48.00	44.50	0.601	46.00
Q1–Q3	29.00–60.00	26.25–60.75		26.50–60.50
	n = 25	n = 78		n = 103
Onset				
Acute and subacute	23 (95.8%)	67 (87.0%)	0.452	90 (89.1%)
Progressive	1 (4.2%)	10 (13.0%)		11 (10.9%)
	*n* = 24	*n* = 77		*n* = 101
CNS involvement				
Meningitis	4 (16.7%)	5 (6.4%)	0.1812	9 (8.8%)
Meningoencephalitis	13 (54.2%)	55 (70.5%)		68 (66.7%)
Meningoencephalomyelitis	7 (29.2%)	18 (23.1%)		25 (24.5%)
	*n* = 24	*n* = 78		*n* = 102
Tumor revealed at screening				
No	22 (88.0%)	64 (83.1%)	0.755	86 (84.3%)
Yes	3 (12.0%)	13 (16.9%)		16 (15.7%)
	*n* = 25	*n* = 77		*n* = 102
Lower limb deficit				
No	12 (48.0%)	50 (67.6%)	0.131	62 (62.6%)
Yes	13 (52.0%)	24 (32.4%)		37 (37.4%)
	*n* = 25	*n* = 74		*n* = 99
Areflexia				
No	17 (68.0%)	37 (88.1%)	0.059	54 (80.6%)
Yes	8 (32.0%)	5 (11.9%)		13 (19.4%)
	n = 25	n = 42		n = 67
Bladder dysfunction				
No	8 (32.0%)	43 (59.7%)	**0.031**	51 (52.6%)
Yes	17 (68.0%)	29 (40.3%)		46 (47.4%)
	*n* = 25	*n* = 72		*n* = 97
Maximal mRS score				
3–6	18 (72.0%)	66 (84.6%)	0.234	84 (81.6%)
0–2	7 (28.0%)	12 (15.4%)		19 (18.4%)
	*n* = 25	*n* = 78		*n* = 103
mRS at last follow-up				
0–2	19 (82.6%)	54 (85.7%)		73 (84.9%)
3–6	4 (17.4%)	9 (14.3%)	0.740	13 (15.1%)
	*n* = 23	*n* = 63		*n* = 86
Duration of follow-up, months				
Median	12.00	14.50	0.084	12.00
Q1–Q3	4.00–12.00	4.25–25.75		4.00–23.00
	*n* = 23	*n* = 62		*n* = 85

CNS, central nervous system; PNS, peripheral nervous system; mRS, modified Rankin Scale

Bold letters indicate a statistically significant difference

### Representative case (Patient 2)

A 33-year-old woman presented to the emergency room with an intense headache associated with fever. The same day, she got confused and her awareness decreased, requiring admission to the ICU. A CSF analysis showed 171 white cells/mm^3^ with 99% of lymphocytes, an elevated protein level of 1.49 g/L, and a normal glucose level (Table 2). A brain MRI showed a leptomeningeal and acoustic–facial bundle contrast enhancement (Fig. 3). An electroencephalogram did not show any epileptic activity. An autoimmune meningo-encephilitis was considered, after negative infectious results, and the patient was treated with intravenous corticosteroids (methylprednisolone 500 mg/day for 3 days). This attack treatment was effective on headache and confusion, and the patient was transferred to a neurology department 5 days after ICU admission. Oral corticosteroids (1 mg/kg/day) were prescribed as maintenance therapy. In the neurology department, she complained of bilateral paresthesia in the feet, clinical examination showed a proximal weakness of the lower limbs associated with abolished deep tendon reflexes, and urinary retention was noticed. The ENMG revealed a bilateral L5/S1 radiculopathy (Table 3). A spinal cord MRI showed a leptomeningeal and lumbosacral root contrast enhancement, without myelitis (Fig. 3). A second CSF analysis was performed and was remarkable for GFAP-Abs. At this time, she walked with a rollator. A GFAP-related meningoencephalitis associated with lower limb radiculitis was finally retained, and the patient was treated with associated intravenous immunoglobulin (IVIg; 0.4 g/kg/day for 5 days). Oral corticosteroids were decreased progressively for 6 months and then stopped. After 9 months, she was able to walk without aid but complained of urinary incontinence and attention deficiency.

## Discussion

We report herein the largest cohort of patients with PNS involvement associated with CSF GFAP-Abs. The following data were instructive: (1) a prevalence of around one-fifth of PNS involvement, indicating that it is not a rare feature; (2) the clinical presentation of PNS involvement was heterogeneous; (3) cranial nerve involvement and radiculitis were the most common presentations; (4) bladder dysfunction was frequent; (5) PNS involvement was rarely the first manifestation; (6) all patients except one had associated CNS involvement; (7) all patients had pleocytosis and elevated protein in CSF; (8) onset was acute or subacute in most cases, and the prognosis seemed favorable.

The peripheral clinical picture in patients with GFAP-Abs is heterogeneous and dominated by cranial nerve involvement and radiculopathy, while inflammatory demyelinating polyradiculoneuropathy, sensory neuronopathy, or length-dependent axonal polyneuropathy were more rarely found. Radiculopathy was found to especially affect the lower limbs and cause proximal motor weakness with frequent root gadolinium enhancement.

These findings are partially consistent with a recently published cohort of patients with CSF GFAP-Abs and PNS involvement, which included 12 patients in whom the PNS involvement was electrophysiologically confirmed [12]. These patients mainly had lower limbs motor weakness with reduced compound muscle action potentials (7/12) on ENMG leading the authors to conclude to predominant motor axonal neuropathy. However, F waves were prolonged in 8/12 patients, questioning the possibility of radiculopathy rather than axonal motor neuropathy [12]. The present findings are also partially consistent with the first published case series of patients with GFAP-Abs and PNS involvement in which acute/subacute inflammatory demyelinating polyradiculoneuropathy was diagnosed in three of six patients [9]. This could be explained by the diagnostic criteria used in the above-mentioned study, as the diagnosis of acute/subacute inflammatory demyelinating polyradiculoneuropathy relied only on prolonged or absent F waves in two of these three cases, which may have instead been considered as cases of radiculopathy or polyradiculopathy in the current study. However, the third case had undoubtful inflammatory demyelinating polyradiculoneuropathy with electrophysiological arguments for peripheral nerve demyelination with prolonged distal latencies and slowed conduction velocities, and segmental demyelination on nerve biopsy [9]. In the cohort presented herein, only one patient fulfilled the criteria of inflammatory demyelinating polyradiculoneuropathy, characterized by a proximal motor weakness of the lower limbs, sensory ataxia, and clear demyelinating abnormalities on ENMG [18]. A direct role of the GFAP-Abs could be suspected in the cases of demyelinating polyradiculoneuropathy, because GFAP is expressed by immature Schwann cells, and demyelinating features were observed in the nerve biopsies of patients with GFAP-Abs expressing this phenotype [6, 9]. The clinicians should, therefore, consider testing CSF GFAP-Abs in cases of atypical CIDP-like diseases associated with CNS symptoms and CSF pleocytosis.

Interestingly, one patient herein had sensory neuronopathy. To our knowledge, it is the first case of sensory neuronopathy associated with GFAP-Abs (although previously reported by Gravier-Dumonceau et al.) [14]. We suggest a direct causal link between the identified CSF GFAP-Abs and such phenotype for the following reasons: (1) GFAP was found to be expressed in the DRG, and a patient’s CSF was able to stain the DRG by immunofluorescence [4, 9], (2) autoimmune processes are frequent in sensory neuronopathies, and several other antibodies have already been described in this syndrome such as Hu, CV2/CRMP5, FGFR3, or AGO antibodies [20–23], (3) the patient presented herein had a lung adenocarcinoma found on tumor screening, suggesting a possible immune paraneoplastic origin [23]. The case reported herein thus suggests that CSF GFAP-Abs should be added to the screening tests of acute/subacute sensory neuronopathy, particularly when a CNS symptom is associated.

Regarding cranial nerves, nerve VI was the most frequently affected. Cranial nerve involvement could also be due to an inflammatory process as suggested by the cranial nerve gadolinium enhancement observed in around one-third of the patients herein. However, nerve VI lesions may not always rely on an autoimmune inflammatory mechanism. Indeed, this cranial nerve is known to be highly sensitive to increased intracranial pressure, and intracranial hypertension is described in patients with CSF GFAP-Abs [14, 24]. Moreover, papilledema, a common feature of intracranial hypertension, was found in four of the patients with nerve VI involvement described herein (40%). None of these patients benefited from CSF pressure measurement. While a direct link between GFAP-Abs and PNS involvement can be suggested in patients with sensory neuronopathy or demyelinating polyradiculoneuropathy, it is more difficult to explain such a link in patients with isolated cranial nerve involvement or radiculitis. Indeed, in such cases, the PNS involvement could be due to a contiguous consequence of the inflammation of brainstem and spinal cord leptomeninges where the roots originate.

Another important finding is the possibility that PNS involvement might be the first manifestation of the disease, prior to CNS symptoms. However, this situation was rare in the present cohort, occurring in 3/25 patients (12%) with PNS involvement. In contrast, five of six patients in a previously published cohort of patients with GFAP-Abs and PNS involvement had inaugural peripheral nerve symptoms [9]. The onset was acute or subacute in all except one of the patients presented herein, which is in line with the previous cohort [9].

The comparison of patients with CSF GFAP-Abs and PNS involvement to those with isolated CNS involvement showed that patients with PNS involvement had more bladder dysfunction probably due to cauda equina impairment. Areflexia tended to be more frequent in the patients with PNS involvement, but the difference was not statistically significant. It could be explained by a lack of data in the group with isolated CNS involvement, where the frequency of areflexia is expected to be low. Interestingly, the type of CNS involvement, especially the frequency of myelitis, was similar in both groups. Finally, the demographic characteristics of the patients with PNS involvement herein were concordant with those of previously published cohorts of GFAP-Abs patients, in which the median age at onset ranged from 44 to 54 and affected between 43 and 68% of female patients [16–18, 25, 26].

We believe that CSF analysis is a decisive tool in the diagnosis of suspected GFAP-Abs-related disease, as pleocytosis and elevated protein level were identified in all the patients herein. In line with previous data, we consider that the absence of pleocytosis and high protein level should lead clinicians to rule out the diagnosis of GFAP-Abs-related disease [14]. Also, root or/and leptomeningeal contrast enhancement was frequently present in patients with abnormal ENMG (81%). Such a radiological pattern should lead to consider the diagnosis [12].

In the present cohort, the 7/25 patients with PNS involvement and CSF GFAP-Abs who were concomitantly tested in the serum all had a negative CBA test. It might be considered unusual for patients with PNS disease to be serum negative and CSF positive regarding Abs detection. However, the pathogenicity of GFAP-Abs is not established, and the patients herein mainly had involvement of proximal parts of the PNS (roots and cranial nerves), that pass through the CSF and leptomeninges. In the previous cohorts, 4/6 and 17/21 patients with PNS involvement had GFAP-Abs detected in serum, but all were concomitantly positive for GFAP-Abs in the CSF when available (19 patients) [9, 12]. Of note, serum herein was tested only by CBA, which is the more specific test but for which the exact sensitivity is unknown. In light of these heterogenous data, it is difficult to draw conclusions about the clinical relevance of GFAP-Abs in the serum of patients with PNS involvement.

Three patients of the present cohort diagnosed with radiculopathy were found to have positive CSF tests for infectious diseases, known to cause root injuries (VZV infection and Lyme disease). We concede that these findings may represent important confounding factors. However, we point out that these infectious agents might have played a role in triggering GFAP-Abs-mediated autoimmunity, in addition to their own pathogenicity. Indeed, in the present study, the clinicians in charge of the patients concluded to a triggering role of infections for a GFAP-Abs-related disease in two of these patients. In a previous study by our group, infectious prodromal symptoms were found in 82% of cases, suggesting a triggering role of infections in this autoimmune disease [14]. A severe axonal sensorimotor neuropathy associated with GFAP-Abs encephalitis and Epstein–Barr virus (EBV) DNA in the CSF has been reported, which the authors hypothesized to be a possible trigger for the autoimmune response [11]. Finally, it is well established that infections can trigger some other neurological autoimmune diseases, sometimes by molecular mimicry between antigens, as in Guillain–Barré syndrome following *Campylobacter Jejuni* or EBV/CMV infections [27], or in NMDAR-Abs encephalitis after herpes simplex virus infection [28, 29]. Regarding the patient with positive VZV PCR in the CSF, he was immunocompetent, did not develop a rash, and presented an undoubtful positive CBA test for GFAP-Abs. The only other patient with a possible alternative cause of PNS involvement was the patient with a history of type 2 diabetes mellitus. However, this patient first developed a central meningitis with fever and headache, and then developed binocular diplopia, proprioceptive ataxia with lower limb apallesthesia, proximal lower limb weakness, and radiculalgia suggestive of PNS involvement. Moreover, the CSF analysis revealed a meningitis and oligoclonal bands. Type 2 diabetes mellitus is not an etiology for sensory neuronopathy. Taken together, these data are not in favor of a PNS involvement due to the type 2 diabetes.

The prognosis of patients with GFAP-Abs and PNS involvement seems to be favorable [12]. In the present cohort, the last follow-up mRS was similar in both patients with or without PNS involvement, suggesting that the PNS involvement does not worsen the prognosis. Moreover, improvement of peripheral signs and ENMG was frequent during follow-up. However, severe sequelae might also persist over months in rare cases of severe neuropathy, as reported in the other studies [11, 12, 18]. An early diagnosis, allowing an early immunosuppressive treatment, is probably an important factor for PNS prognosis.

The present study has some limitations. First, it is a retrospective study, but detailed clinical data were provided by the clinicians in charge of the patients using a structured questionnaire. Then, GFAP-Abs testing was performed in patients recruited by the two French reference centers for CNS inflammatory diseases who were mainly patients presenting with signs of encephalitis or myelitis. Patients presenting with isolated suspected inflammatory PNS involvement in the same time window were mainly not tested for GFAP-Abs, causing a possible selection bias and underestimation of GFAP-Abs in these patients.

In conclusion, this retrospective cohort study confirmed that PNS involvement is not a rare feature in GFAP-Abs-related disease. Patients typically present with acute or subacute cranial nerve injury and/or lower limb radiculopathy. These manifestations are mainly associated with CNS symptoms but may, in rare cases, present initially as isolated. Bladder dysfunction is an important clue suggesting a PNS involvement. In future prospective studies, screening GFAP-Abs in patients with inflammatory PNS disease of unknown etiology, associated or not with CNS symptoms or meningitis, may be useful to better define the spectrum of GFAP-Abs-related disease phenotypes.

### Supplementary Information

Below is the link to the electronic supplementary material.

Supplementary file1 (DOCX 33 kb)

## Data Availability

Anonymized data will be shared upon request from any qualified investigator for purposes of replicating procedures and results.
